# Impact of Computed Tomography-Based, Artificial Intelligence-Driven Volumetric Sarcopenia on Survival Outcomes in Early Cervical Cancer

**DOI:** 10.3389/fonc.2021.741071

**Published:** 2021-09-24

**Authors:** Qingling Han, Se Ik Kim, Soon Ho Yoon, Taek Min Kim, Hyun-Cheol Kang, Hak Jae Kim, Jeong Yeon Cho, Jae-Weon Kim

**Affiliations:** ^1^ Department of Obstetrics and Gynecology, Seoul National University College of Medicine, Seoul, South Korea; ^2^ Department of Radiology, UMass Memorial Medical Center, Worcester, MA, United States; ^3^ Department of Radiology, Seoul National University College of Medicine, Seoul, South Korea; ^4^ Department of Radiation Oncology, Seoul National University College of Medicine, Seoul, South Korea

**Keywords:** uterine cervical neoplasms, body composition, sarcopenia, muscles, abdominal fat, prognosis, survival

## Abstract

The purpose of this study was to investigate the impact of sarcopenia and body composition change during primary treatment on survival outcomes in patients with early cervical cancer. We retrospectively identified patients diagnosed with 2009 International Federation of Gynecology and Obstetrics stage IB1-IIA2 cervical cancer who underwent primary radical hysterectomy between 2007 and 2019. From pre-treatment CT scans (n = 306), the skeletal muscle area at the third lumbar vertebra (L3) and the waist skeletal muscle volume were measured using an artificial intelligence-based tool. These values were converted to the L3 and volumetric skeletal muscle indices by normalization. We defined L3 and volumetric sarcopenia using 39.0 cm^2^/m^2^ and the first quartile (Q1) value, respectively. From pre- and post-treatment CT scan images (n = 192), changes (%) in waist skeletal muscle and fat volumes were assessed. With the use of Cox regression models, factors associated with progression-free survival (PFS) and overall survival (OS) were analyzed. Between the L3 sarcopenia and non-sarcopenia groups, no differences in PFS and OS were observed. In contrast, volumetric sarcopenia was identified as a poor prognostic factor for PFS (adjusted hazard ratio [aHR], 1.874; 95% confidence interval [CI], 1.028–3.416; *p* = 0.040) and OS (aHR, 3.001; 95% CI, 1.016–8.869; *p* = 0.047). During primary treatment, significant decreases in waist skeletal muscle (median, −3.9%; *p* < 0.001) and total fat (median, −5.3%; *p* < 0.001) were observed. Of the two components, multivariate analysis revealed that the waist fat gain was associated with worse PFS (aHR, 2.007; 95% CI, 1.009–3.993; *p* = 0.047). The coexistence of baseline volumetric sarcopenia and waist fat gain further deteriorated PFS (aHR, 2.853; 95% CI, 1.257–6.474; *p* = 0.012). In conclusion, baseline volumetric sarcopenia might be associated with poor survival outcomes in patients with early cervical cancer undergoing primary RH. Furthermore, sarcopenia patients who gained waist fat during primary treatment were at a high risk of disease recurrence.

## Introduction

Cervical cancer is a major health problem, as it ranks the fourth highest incidence and mortality rates among cancers in women worldwide (1). The incidence of cervical cancer shows a geographical difference. Age-standardized incidence rate of cervical cancer is higher in Korea than in the United States and other Western countries (2, 3). However, owing to the effective cervical cancer screening program, more than half (55.8%) of cervical cancer cases are diagnosed at a localized disease in Korea (4, 5). For early cervical cancer, primary radical hysterectomy (RH) is recommended as one of the standard treatment options (6, 7).

Body composition analysis refers to quantifying different body compartments, such as fat and muscle, and assessing their relative ratio in an individual. Researchers have mainly focused on excessive fat accumulation, so-called obesity, and they investigated the relationship between obesity and risk of developing cancer (8, 9) and the role of obesity in cancer survival and recurrence (10, 11). Sarcopenia, characterized by the loss of skeletal muscle mass and function, recently emerged in the cancer research field, as it was associated with higher recurrence and mortality rates, surgical complications, and treatment-related toxicity (12–15). In cervical cancer, only few studies have investigated prognostic role of pre-treatment sarcopenia, resulting in conflicting results (16–18). Moreover, all these previous studies included patients who underwent primary concurrent chemoradiation therapy (CCRT) or radiation therapy (RT), rather than primary RH.

For body composition analysis, computed tomography (CT) is widely used because it can quantify the body composition components. Researchers have measured individuals’ area of skeletal muscle and adipose tissue at the third lumbar vertebral body (L3)-level cross-sectional image of CT scans, which is known to reflect amounts of total body muscle and adipose tissue well (19, 20). In addition, the latest high-throughput technology allows automated and fast volumetric measurements of each component from CT scans (21, 22). With the use of such an advanced tool, tracking the volumetric change of specific body composition components is feasible (23), which has not yet been investigated in early cervical cancer.

Thus, we aimed to investigate the impact of pre-treatment sarcopenia determined by two different measurements (L3 level skeletal muscle area and waist skeletal muscle volume) on survival outcomes in Korean patients with early-stage cervical cancer who underwent primary RH. Additionally, we traced the change of body composition during primary treatment and investigated their prognostic roles.

## Materials and Methods

### Study Population

From the institution’s cervical cancer cohort database, we identified and collected patients who met the following conditions: 1) patients aged 20 years or older at the time of diagnosis; 2) patients diagnosed with 2009 International Federation of Gynecology and Obstetrics (FIGO) stage IB1 to IIA2 cervical cancer who were treated at Seoul National University Hospital between January 2007 and December 2019; 3) patients who underwent primary type B-C RH, according to Querleu–Morrow classification (24), and pelvic lymphadenectomy by faculty who finished gynecologic oncology fellowship; and 4) those whose pre-treatment CT scans, performed less than a month before the primary surgery, were stored in the Picture Archiving and Communication System.

Meanwhile, patients with the following conditions were excluded: 1) those who received neoadjuvant chemotherapy prior to RH; 2) those whose tumor had histologic types other than squamous cell carcinoma, usual type adenocarcinoma, and adenosquamous carcinoma; 3) those who were diagnosed with other cancers before and/or at the time of cervical cancer diagnosis; 3) those with insufficient clinicopathologic data; 4) those lost to follow-up before completion of primary treatment; and 5) those for whom we were unable to obtain pre-treatment CT scans.

**Figure 1:**
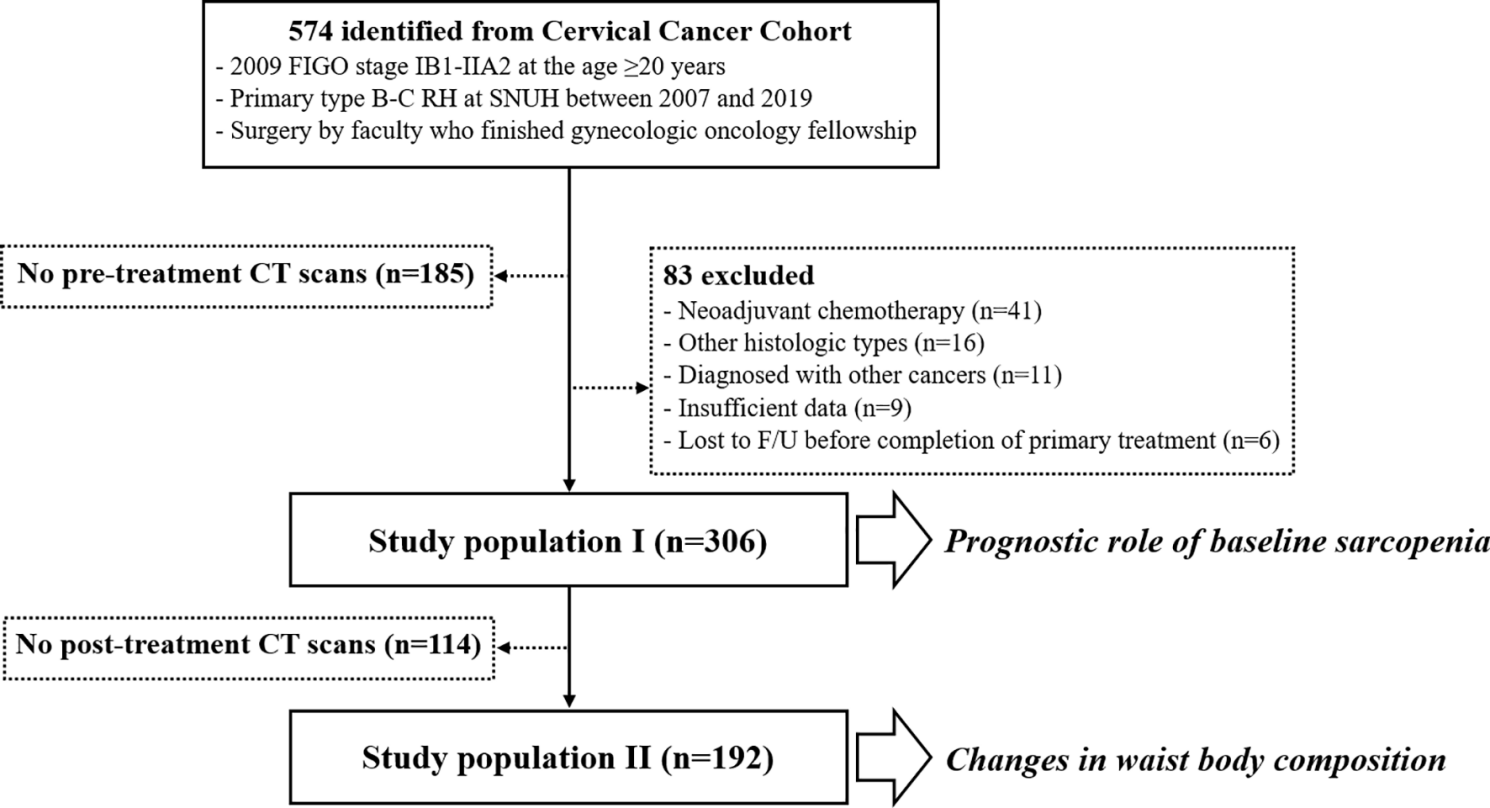
Flow diagram depicting selection of study population.

In total, 306 patients were included in this analysis (study population I). To assess changes in body composition components, we further identified 192 patients whose post-treatment CT scans were available (study population II). For the patients who did not undergo adjuvant treatment, we referred to CT scans obtained 3 months after the surgery. For the patients who received adjuvant RT or CCRT, we used CT scans obtained within a month after the completion of RT (**Figure 1**).

### Data Collection

We collected patients’ clinicopathologic features, such as age at diagnosis, FIGO stage, surgical approach, histologic type, radicality of hysterectomy, para-aortic lymphadenectomy, pathologic risk factors, risk group, and adjuvant treatment. Based on pre-treatment body mass index (BMI), patients were divided into four groups, according to the WHO’s recommendation for Asian population (25): <18.5 kg/m^2^ (underweight), 18.5–22.9 kg/m^2^ (normal), 23.0–24.9 kg/m^2^ (overweight), and ≥25.0 kg/m^2^ (obese). Clinical cervical tumor size was determined by either colposcopic examination or pre-treatment magnetic resonance imaging (MRI).

During pre-treatment workup, advanced imaging modalities, such as MRI and whole-body ^18^F-FDG positron emission tomography (PET)/CT imaging, have been frequently conducted at this institution. While we measure the cervical tumor size and evaluate parametrium involvement using MRI, we evaluate distant site metastasis using CT scans and PET/CT imaging. Pelvic and para-aortic lymph node status is assessed from all the available imaging modalities. Among the study population (n = 306), 15 (4.9%) received pre-treatment CT scans only, while 36 (11.8%) and 63 (20.6%) received CT scans plus MRI and CT scans plus PET/CT imaging, respectively. The other 192 (62.7%) patients received all three imaging modalities.

After surgery, patients who had lymph node metastasis, positive resection margins, or parametrium involvement were classified as the high-risk group. According to the Sedlis criteria, we classified patients with various combinations of the three factors (tumor size, depth of invasion, and lymphovascular space invasion) as the intermediate-risk group (26). High-risk and intermediate-risk patients received adjuvant CCRT or RT after RH.

Adjuvant RT consisted of a combination of external beam RT (EBRT) with/without high-dose-rate intracavitary radiotherapy (HDR-ICR). With regard to RT planning, our institution had used 3D conformal RT before November 2015 and adopted intensity-modulated RT (IMRT) since then. The prescribed dose fractionation schedule for pelvic EBRT was 50.4 Gy in 28 fractions. For patients with pathologically confirmed para-aortic lymph node metastasis, extended field RT consisting of an additional boost dose of 9–10 Gy in five fractions to the para-aortic lymphatics was delivered. HDR-ICR was implemented with the dose fractionation schedule of 15 Gy in three fractions. The treatment duration of RT usually took 5–6 weeks. As the most common regimen for CCRT, 40 mg/m^2^ of cisplatin was administered weekly for 4–6 cycles during EBRT.

From the patients’ medical records, we also collected gastrointestinal toxicities that occurred during adjuvant RT. Because of the retrospective study design, it was challenging to identify the exact grade according to the Common Terminology Criteria for Adverse Events version 5.0 (27). Instead, we checked the presence or absence of any grade gastrointestinal toxicities.

Surveillance frequency for symptom review and examination depended on FIGO stage, pathologic risk factors, and adjuvant therapy (6, 7). In general, patients who completed the initial treatment (hysterectomy and adjuvant treatment) consulted a physician every 3 months in the first 2 years, and every 6 months for the next 3 years. Thereafter, patients visited the clinic every year.

We determined the progression or recurrence of the disease from imaging studies based on the Response Evaluation Criteria in Solid Tumors version 1.1 (28). Progression-free survival (PFS) refers to the time interval between the beginning of treatment and disease progression. Overall survival (OS) was defined as the time interval between the date of diagnosis and the date of cancer-related death or the last visit.

### Imaging Analysis

Imaging analysis methods for this study were the same as our previous study on patients with epithelial ovarian cancer (23), including the use of the same commercially available, artificial intelligence-based software (DEEPCATCH v1.0.0.0; MEDICALIP Co. Ltd., Seoul, Korea). In brief, we used this deep neural network-based software for automatic volumetric segmentation of body composition (skeletal muscle, abdominal visceral fat, and subcutaneous fat) from anonymized, precontrast CT images in DICOM format. According to the previous validation study, the software’s average segmentation accuracy was reported as 97% compared with manual segmentation (21). After segmentation, the abdominal waist was automatically labeled based on WHO’s waist definition (29): between the lower end of the thoracic ribs and the upper end of the iliac crest. One expert radiologist (SHY) confirmed the results of automatic segmentation and labeling. Subsequently, the waist volume (cm^3^) of skeletal muscle and total fat (sum of abdominal visceral fat and subcutaneous fat) were quantified and normalized to the height (m^3^), generating the volumetric skeletal muscle index (SMI) and total fat index. This software also automatically measured skeletal muscle area (cm^2^) from the single cross-sectional CT image at the L3 level. The skeletal muscle area was normalized to the height (m^2^) and reported as the L3 SMI (**Supplementary Figure 1**).

### Statistical Analysis

L3 sarcopenia was defined as an individual’s L3 SMI <39.0 cm^2^/m^2^, per the cutoff value proposed by an international consensus of cancer cachexia (30). This value was also used in our previous study, which investigated the impact of sarcopenia on survival outcomes in patients with advanced-stage high-grade serous ovarian cancer (31). Because there is no study of volumetric SMI, we used the Q1 value and divided patients into volumetric sarcopenia and non-sarcopenia groups accordingly.

Differences in the pre- and post-treatment waist volume of body compositions components were evaluated using the paired t-test. Change (%) in a specific component was calculated as follows:


$$\frac{x_{Post-treatment}-x_{Pre-treatment}}{x_{Pre-treatment}}\times 100$$

We regarded a negative value as a loss during treatment. The extent of changes in body composition components between the two groups was compared using Student’s t-test, while that among the three or more groups were compared using one-way ANOVA.

The characteristics and survival outcomes were compared between the two groups, such as volumetric sarcopenia *versus* non-sarcopenia groups. We used Student’s t-test or Mann–Whitney U test to compare continuous variables, and Pearson’s chi-square test or Fisher’s exact test to compare categorical variables. Pearson’s correlation coefficient test was used to calculate the correlation value. Kaplan–Meier methods and log-rank tests were used for the survival analysis. In multivariate analysis, we used the Cox proportional hazards model to calculate adjusted hazard ratios (aHRs) and 95% confidence intervals (CIs). IBM SPSS software (version 25.0; IBM Corp., Armonk, NY, USA) was used for statistical analysis. We considered a *p*-value <0.05 as statistically significant.

### Ethics Statement

This study was approved by the Institutional Review Board of Seoul National University Hospital (No. H-2012-061-117) and performed according to the principles of the Declaration of Helsinki. The requirement for informed consent was waived.

## Results

### Patients’ Characteristics

**Table 1** describes the clinicopathologic features of the study population I (n = 306). Squamous cell carcinoma was the most common histological type (74.2%), and 64.1% of the patients had 2009 FIGO stage IB1. The median clinical cervical tumor size was 26.5 mm (interquartile range [IQR], 10.0–40.1). After RH, 119 (38.9%) did not undergo adjuvant treatment, while 30 (9.8%) and 157 (51.3%) underwent adjuvant RT and CCRT, respectively.

**Table 1:** Clinicopathologic characteristics of volumetric sarcopenia and non-sarcopenia groups.

Characteristics	All (n = 306, %)	Volumetric sarcopenia (n = 76, %)	Volumetric non-sarcopenia (n = 230, %)	*p*
Age, years				
Mean ± SD	51.5 ± 11.3	52.6 ± 11.0	51.1 ± 11.4	0.295
BMI, kg/m^2^				
Median (IQR)	23.4 (21.2−25.9)	22.1 (20.2−24.7)	23.9 (21.7−26.4)	<0.001
Underweight (<18.5)	12 (3.9)	6 (7.9)	6 (2.6)	0.001
Normal (18.5–22.9)	132 (43.1)	42 (55.3)	90 (39.1)	
Overweight (23.0–24.9)	58 (19.0)	15 (19.7)	43 (18.7)	
Obesity (≥25.0)	104 (34.0)	13 (17.1)	91 (39.6)	
Surgical approach				0.914
Open	143 (46.7)	37 (48.7)	106 (46.1)	
Laparoscopy	131 (42.8)	31 (40.8)	100 (43.5)	
Robot-assisted surgery	32 (10.5)	8 (10.5)	24 (10.4)	
Conization	88 (28.8)	17 (22.4)	71 (30.9)	0.156
Histologic type				0.209
Squamous cell carcinoma	227 (74.2)	62 (81.6)	165 (71.7)	
Adenocarcinoma	66 (21.6)	11 (14.5)	55 (23.9)	
Adenosquamous carcinoma	13 (4.2)	3 (3.9)	10 (4.3)	
2009 FIGO stage				0.293
IB1	196 (64.1)	47 (61.8)	149 (64.8)	
IB2	49 (16.0)	9 (11.8)	40 (17.4)	
IIA1	21 (6.9)	8 (10.5)	13 (5.7)	
IIA2	40 (13.1)	12 (15.8)	28 (12.2)	
Radicality of hysterectomy				0.285
Type B	27 (8.8)	9 (11.8)	18 (7.8)	
Type C	279 (91.2)	67 (88.2)	212 (92.2)	
Para-aortic lymphadenectomy				0.916
No	220 (71.9)	55 (72.4)	165 (71.7)	
Sampling/dissection	86 (28.1)	21 (27.6)	65 (28.3)	
Clinical cervical tumor size^*^, mm				
Median (IQR)	26.5 (10.0−40.1)	26.5 (13.5−26.5)	26.5 (10.0−40.0)	0.839
<20	109 (35.6)	26 (34.2)	83 (36.1)	0.897
≥20 and <40	110 (35.9)	29 (38.2)	81 (35.2)	
≥40	87 (28.4)	21 (27.6)	66 (28.7)	
Pathologic risk factors				
Parametrial invasion	62 (20.3)	18 (23.7)	44 (19.1)	0.392
Lymph node metastasis	85 (27.8)	21 (27.6)	64 (27.8)	0.974
Resection margin involvement	30 (9.8)	7 (9.2)	23 (10.0)	0.841
LVSI	154 (50.3)	36 (47.4)	118 (51.3)	0.552
Deep one-third stromal invasion	161 (52.6)	39 (51.3)	122 (53.0)	0.794
Risk group				0.735
Low risk	119 (8.9)	30 (39.5)	89 (38.7)	
Intermediate risk	70 (22.9)	15 (19.7)	55 (23.9)	
High risk	117 (38.2)	31 (40.8)	86 (37.4)	
Adjuvant treatment				0.788
No	119 (38.9)	29 (38.2)	90 (39.1)	
RT only	30 (9.8)	9 (11.8)	21 (9.1)	
CCRT	157 (51.3)	38 (50.0)	119 (51.7)	

BMI, body mass index; CCRT, concurrent chemoradiation therapy; FIGO, International Federation of Gynecology and Obstetrics; IQR, interquartile range; LVSI, lymphovascular space invasion; RT, radiation, therapy; SD, standard deviation.

^*^Measured by either colposcopic examination or pre-treatment magnetic resonance imaging.

Of 187 patients with (CC)RT, 154 (82.4%) and 33 (17.6%) received EBRT and EBRT plus HDR-ICR, respectively (**Supplementary Table 1**). For RT planning, 3D conformal RT was conducted in 116 (62.0%), whereas IMRT was conducted in 71 (38.0%). Extended field RT was administered in nine (4.8%) patients. Five patients refused RT due to poor general condition (early termination of RT). During RT, more than a half (55.1%) experienced nausea. Other common gastrointestinal toxicities were as follows: diarrhea (45.5%), constipation (31.6%), anorexia (22.5%), abdominal pain (20.3%), and vomiting (19.3%) (in the order of frequency).

In terms of baseline body composition, the median values for L3 SMI and volumetric SMI were 39.4 cm^2^/m^2^ (IQR, 34.0–44.3) and 206.5 cm^3^/m^3^ (IQR, 181.5–236.2), respectively. As shown in **Supplementary Figure 2**, baseline BMI was weakly correlated with L3 SMI (Pearson’s correlation coefficient r = 0.249; *p* < 0.001) and volumetric SMI (r = 0.423; *p* < 0.001). The L3 SMI and volumetric SMI also showed a very weak positive correlation (r = 0.176; *p* = 0.002).

During a median observation period of 55.2 months, 50 (16.3%) patients experienced disease recurrence, and 14 (4.6%) patients died.

### Prognostic Role of Baseline Sarcopenia

**Figure 2:**
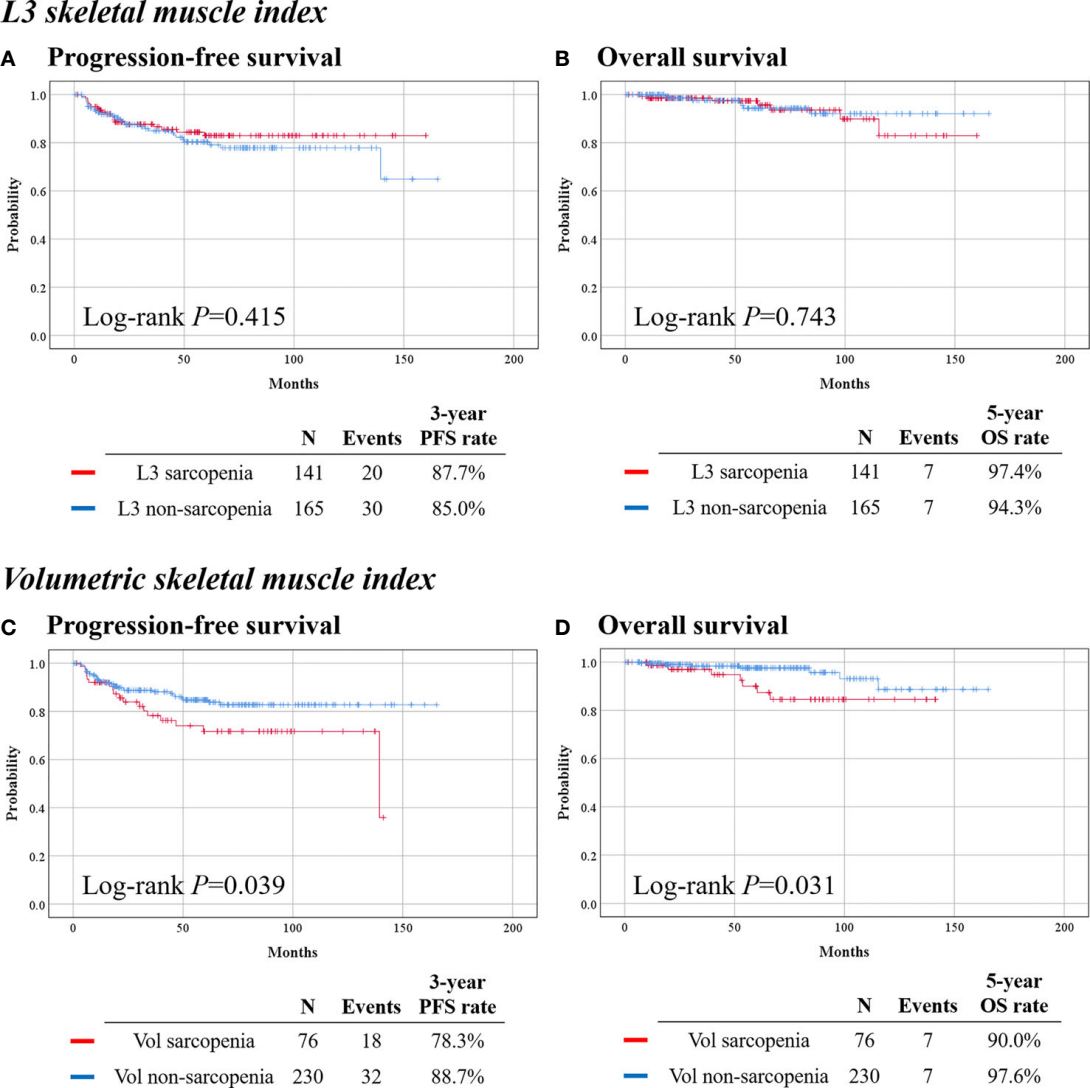
Survival outcomes of patients by skeletal muscle index. (Top) Calculated from L3 level cross-sectional image. (Bottom) Calculated from volumetric measurement of the waist. **(A, C)** Progression-free survival. **(B, D)** Overall survival.

With the use of the well-known cutoff value (39.0 cm^2^/m^2^) of L3 SMI, 141 (46.1%) and 165 (53.9%) patients were assigned to the L3 sarcopenia and non-sarcopenia groups, respectively. Patients in the L3 sarcopenia group had a significantly lower BMI (mean, 22.1 *vs*. 24.8 kg/m^2^; *p* < 0.001), than had the L3 non-sarcopenia group. However, other clinicopathologic characteristics were similar between the two groups (**Supplementary Table 2**). In survival analysis, the L3 sarcopenia and non-sarcopenia groups showed similar PFS (*p* = 0.415) and OS (*p* = 0.743) (**Figures 2A, B**).

With the use of the Q1 value (181.5 cm^3^/m^3^) of volumetric SMI, 76 (24.8%) and 230 (75.2%) were identified as the volumetric sarcopenia and non-sarcopenia groups, respectively. The volumetric sarcopenia group had a significantly lower BMI (22.1 *vs*. 23.9 kg/m^2^; *p* < 0.001) than the volumetric non-sarcopenia group, while other baseline clinicopathologic characteristics were similar between the two groups (**Table 1**).

Among the patients who received (CC)RT (n = 187), the proportions of patients who received HDR-ICR (12.8% *vs*. 19.3%; *p* = 0.310) and extended field EBRT (2.1% *vs*. 5.7%; *p* = 0.454) were also similar between the volumetric sarcopenia and non-sarcopenia groups (**Supplementary Table 1**). However, IMRT was less frequently used in the volumetric sarcopenia group (21.3% *vs*. 43.6%; *p* = 0.006). Regarding incidences of gastrointestinal toxicities during RT, patients in the volumetric sarcopenia group experienced diarrhea (59.6% vs. 40.7%; *p* = 0.025) and vomiting (29.8% *vs*. 15.7%; *p* = 0.034) more frequently, but similar other gastrointestinal toxicities.

In survival analysis, the volumetric sarcopenia group showed significantly worse PFS (3-year PFS rate, 78.3% *vs*. 88.7%; *p* = 0.039) and OS (5-year OS rate, 90.0% *vs*. 97.6%; *p* = 0.031), than the volumetric non-sarcopenia group (**Figures 2C, D**). In multivariate analysis that adjusted for clinicopathologic factors, volumetric sarcopenia was identified as a poor prognostic factor for PFS (aHR, 1.874; 95% CI, 1.028–3.416; *p* = 0.040) and OS (aHR, 3.001; 95% CI, 1.016–8.869; *p* = 0.047) (**Table 2**).

**Table 2:** Factors associated with patients’ survival outcomes.

Characteristics		*Progression-free survival*						*Overall survival*					
		*Univariate analysis*			*Multivariate analysis*			*Univariate analysis*			*Multivariate analysis*		
		HR	95% CI	*p*	aHR	95% CI	*p*	HR	95% CI	*p*	aHR	95% CI	*p*
Age	≥50 *vs*. <50 years	1.159	0.662–2.027	0.606				1.266	0.439–3.654	0.663			
BMI	Continuous	1.010	0.938−1.087	0.789	1.034	0.958−1.117	0.387	0.996	0.859−1.154	0.953			
2009 FIGO stage	IIA *vs*. IB	1.418	0.764–2.632	0.268				2.599	0.898–7.517	0.078			
Histologic type	Non-SCC *vs*. SCC	1.096	0.591–2.032	0.771	1.737	0.903–3.340	0.098	1.084	0.339–3.462	0.892	2.147	0.612–7.533	0.233
Risk group	High *vs*. low intermediate	3.600	1.986−6.522	<0.001	3.882	2.103–7.168	<0.001	4.282	1.340−13.683	0.014	4.739	1.397–16.072	0.013
Adjuvant treatment	Yes *vs*. no	3.934	1.769–8.745	0.001				3.996	0.891–17.923	0.070			
Surgical approach	MIS *vs*. open	1.005	0.575–1.758	0.985				0.729	0.238–2.239	0.581			
Volumetric sarcopenia	Yes *vs*. no	1.823	1.023–3.248	0.042	1.874	1.028–3.416	0.040	3.004	1.052–8.574	0.040	3.001	1.016–8.869	0.047

aHR, adjusted hazard ratio; BMI, body mass index; CI, confidence interval; FIGO, International Federation of Gynecology and Obstetrics; HR, hazard ratio; MIS, minimally invasive surgery; SCC, squamous cell carcinoma.

### Changes in Waist Body Composition

From the study population I, 114 patients were excluded owing to the absence of post-treatment CT scans. Compared with the study population II (n = 192), these 114 patients had significantly smaller clinical cervical tumor size (median, 20.0 *vs*. 30.0 mm; *p* = 0.026) and less frequent lymph node metastasis (20.2% *vs*. 32.3%; *p* = 0.022) and, therefore, omitted adjuvant treatment more frequently (46.5% *vs*. 34.4%; *p* = 0.036) (**Supplementary Table 3**).

Next, we evaluated changes in body composition components among 192 patients in the study population II. **Supplementary Figure 3** depicts the distribution of the patients by extent of changes in body composition components during the treatment: while 65.1% of the patients experienced loss of waist skeletal muscle volume, 61.5% experienced loss of waist total fat volume. There were significant changes in waist skeletal muscle (*p* < 0.001) and total fat (*p* < 0.001) volumes with median values of −3.9% (IQR, −11.0 to 3.7) and −5.3% (IQR, −17.6 to 8.0), respectively. Correlation analyses revealed that there were no correlations between baseline BMI and changes in waist skeletal muscle and total fat volumes (**Supplementary Figures 4A, B**). In contrast, a positive, moderate relationship was observed between the extent of skeletal muscle volume change and that of total fat volume change (r = 0.556; *p* < 0.001) (**Supplementary Figure 4C**).

The extent of skeletal muscle volume change was not associated with patients’ FIGO stage, pathologic risk group, adjuvant treatment, and baseline BMI classification (**Supplementary Table 4**). Meanwhile, the extent of total fat volume change was associated with patients’ FIGO stage (*p* = 0.002) and administration of (CC)RT, rather than no adjuvant treatment (median, −6.1% *vs*. −2.3%; *p* = 0.034). Patients without baseline volumetric sarcopenia showed significantly greater loss of skeletal muscle (median, −4.5% *vs*. 1.2%; *p* = 0.003) and total fat (median, −6.7% *vs*. 6.0%; *p* = 0.011) volumes, than did those with baseline volumetric sarcopenia. Patients who received open RH, rather than minimally invasive RH, also showed significantly greater loss of skeletal muscle (median, −7.5% *vs*. −1.7%; *p* = 0.001) and total fat (median, −12.9% *vs*. 0.2%; *p* < 0.001) volumes.

Among the patients who received (CC)RT (n = 126), the use of IMRT, HDR-ICR, and extended field EBRT was not associated with the extent of changes in skeletal muscle and total fat volumes (**Supplementary Table 5**). Among the various gastrointestinal toxicities during RT, none was associated with the extent of body composition changes in body composition components, except vomiting: patients who experienced vomiting showed significantly greater loss of total fat volume than those who did not (median, −17.1% *vs*. −5.6%; *p* = 0.049).

Next, we focused on prognostic implications of fat gain or loss during cervical cancer treatment. As shown in **Supplementary Table 6**, patients who gained waist total fat volume (n = 74) and those who lost (n = 118) had similar clinicopathologic characteristics, except for surgical approach and para-aortic lymphadenectomy. Patients in the total fat gain group received minimally invasive RH more frequently (66.2% *vs*. 40.7%; *p* = 0.001) and para-aortic lymphadenectomy less frequently (18.9% *vs*. 41.5%; *p* = 0.001), than did those in the total fat loss group.

**Figure 3:**
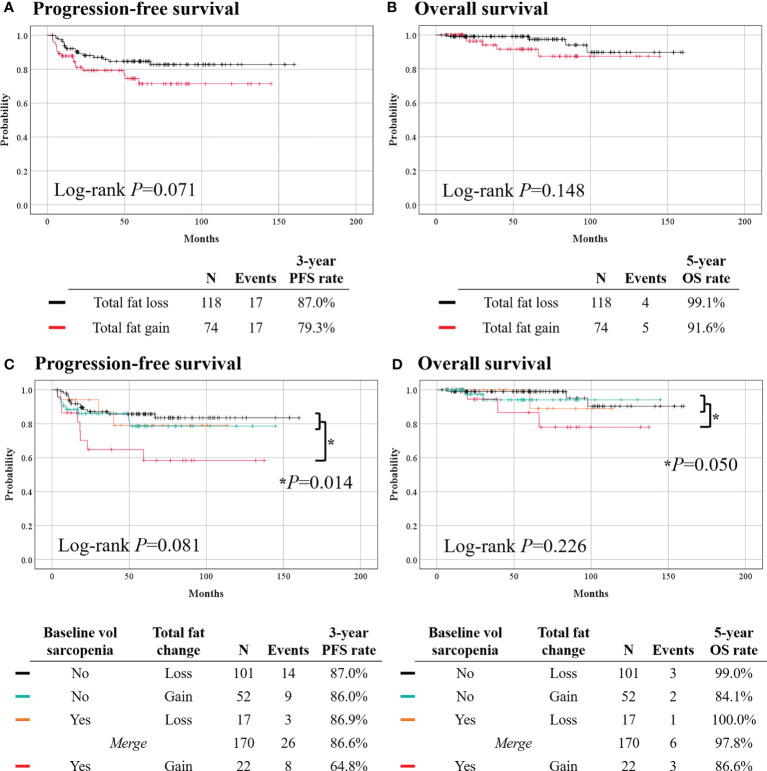
Comparisons of survival outcomes according to changes in total fat volume (top) and combinations of baseline volumetric sarcopenia and waist total fat change (bottom). **(A, C)** Progression-free survival. **(B, D)** Overall survival.

During a median observation period of 55.6 months, no differences in PFS (3-year PFS rate, 79.3% *vs*. 87.0%; *p* = 0.071) and OS (5-year OS rate, 91.6% *vs*. 99.1%; *p* = 0.148) were observed between the total fat gain and loss groups (**Figure 3**). However, in multivariate analyses adjusting for clinicopathologic factors, total fat volume gain was identified as an independent poor prognostic factor for PFS (aHR, 2.007; 95% CI, 1.009–3.993; *p* = 0.047) (**Table 3**). Owing to the small events, we could not conduct further analysis for OS.

**Table 3:** Changes in waist body composition and survival outcomes.

Characteristics		*Progression-free survival*								
		*Univariate analysis*			*Multivariate analysis*			*Multivariate analysis*		
		HR	95% CI	*p*	aHR	95% CI	*p*	aHR	95% CI	*p*
Age	≥50 *vs*. <50 years	1.318	0.665–2.612	0.429						
BMI	Continuous	0.969	0.879–1.069	0.534						
2009 FIGO stage	IIA *vs*. IB	0.951	0.430–2.101	0.900						
Histologic type	Non-SCC *vs*. SCC	2.106	1.053–4.212	0.035	2.699	1.322–5.510	0.006	2.933	1.409–6.104	0.004
Risk group	High *vs*. low intermediate	2.278	1.127–4.603	0.022	2.973	1.415–6.249	0.004	2.773	1.322–5.821	0.007
Adjuvant treatment	Yes *vs*. no	2.462	1.019–5.947	0.045						
Surgical approach	MIS *vs*. open	1.252	0.638–2.459	0.514	1.461	0.715–2.984	0.299	1.556	0.774–3.127	0.214
Total fat change	Gain *vs*. loss	1.840	0.939–3.607	0.076	2.007	1.009–3.993	0.047			
Baseline volumetric sarcopenia and total fat change	Sarcopenia plus fat gain *vs*. others	2.613	1.182–5.776	0.018				2.853	1.257–6.474	0.012

aHR, adjusted hazard ratio; BMI, body mass index; CI, confidence interval; FIGO, International Federation of Gynecology and Obstetrics; HR, hazard ratio; MIS, minimally invasive surgery; SCC, squamous cell carcinoma.

Lastly, we classified patients by the combinations of baseline volumetric sarcopenia and waist total fat change during primary treatment. The baseline volumetric sarcopenia patients who gained total fat (n = 22) showed significantly worse PFS (3-year PFS rate, 64.8% *vs*. 86.6%; *p* = 0.014) than others (n = 170); however, no difference in OS was observed (5-year OS rate, 86.6% *vs*. 97.8%; *p* = 0.050) (**Figure 3**). The two groups had similar clinicopathologic characteristics (**Supplementary Table 7**). Multivariate analyses revealed that initial volumetric sarcopenia with total fat gain during primary treatment was associated with worse PFS (aHR, 2.853; 95% CI, 1.257–6.474; *p* = 0.012) (**Table 3**).

## Discussion

In this study, we found that the pre-treatment or baseline L3 sarcopenia did not affect survival outcomes in patients with early cervical cancer who underwent primary RH. However, patients with volumetric sarcopenia showed significantly higher disease recurrence and mortality, than did those with volumetric non-sarcopenia. Regarding changes in body composition components during primary treatment, the volumetric total fat gain was identified as a poor prognostic factor for PFS.

CT-determined L3 sarcopenia was reported as a poor prognostic factor for many malignancies despite the cutoff values varying among the studies. According to a Korean retrospective study, sarcopenia, defined as L3 SMI ≤31 cm^2^/m^2^ for women and ≤49 cm^2^/m^2^ for men, was an independent poor prognostic factor for OS in patients with advanced gastric cancer (15). Defining sarcopenia as L3 SMI <29.9 cm^2^/m^2^ for women and <49.5 cm^2^/m^2^ for men, Xie et al. reported that baseline sarcopenia was closely related to the risk of recurrence, postoperative complications, and long-term prognosis in Chinese elderly colorectal cancer patients (32).

In contrast, studies conducted in cervical cancer have reported inconsistent results. Yoshikawa et al. measured L3 psoas muscle index (PMI) of Japanese patients with metastatic cervical cancer (n = 40) and identified L3 PMI ≤3.72 cm^2^/m^2^ as an independent poor prognostic factor for OS (16). In contrast, Lee et al. (17) and Matsuoka et al. (18) observed no association between baseline sarcopenia and survival in patients with locally advanced cervical cancer who underwent primary CCRT or RT, similar to our results. However, these two studies differed from our study in terms of ethnicity (Taiwanese *vs*. Japanese *vs*. Korean) and sarcopenia definition (L3 SMI, <41.0 *vs*. <36.55 *vs*. <39.0 cm^2^/m^2^), besides the stage and primary treatment methods.

We recognize that the analysis of a single cross-sectional CT image at the L3 level is a well-established, standard method for body composition analysis. However, this method has limitations. Due to the displacement of the gastrointestinal tract, the abdominal muscle and visceral fat may be measured inaccurately on a single abdominal CT image; the distribution of muscle and visceral fat may vary as high as twice the true value (33). Therefore, a volumetric measurement might be a more accurate method than a single areal measurement. Some might argue that ascites, bowel obstruction, or huge abdominal mass might interfere with accurate volumetric measurement (22). However, such cases were not identified in our study population, as we included only those with early-stage disease.

Compared with L3 SMI, waist volumetric SMI is a relatively new concept; thus, there is no established cutoff value for the volumetric sarcopenia. In this study, we classified patients with volumetric sarcopenia using the Q1 value of the waist volumetric SMI, considering that many early studies on sarcopenia defined cutoff values based on sex-specific, lowest 20% of the study group (34), and recent studies on sarcopenia also use Q1 or quartiles to investigate their impact on cancer prognosis or other health outcomes, such as metabolic syndrome (35, 36). Further population-based studies are warranted to determine an optimal cutoff value for the presence of volumetric sarcopenia.

There are many reasons for decreased skeletal muscle in cancer patients (37). To date, studies on sarcopenia in cancer patients have been conducted in the context of cancer cachexia (38). Patients with cancer cachexia, especially those with enlarging tumor masses, suffer metabolic dysfunction towards catabolism. Considering that the current study population had early-stage disease, influence of cancer cachexia on the pre-treatment sarcopenia seems to be minimal. However, we also admit that even among patients with early cervical cancer, some might already have cancer cachexia at the time of diagnosis. As patients with volumetric sarcopenia were at high risk of disease recurrence in our study, physicians may consider routine baseline body composition analysis to screen for volumetric sarcopenia.

According to the sarcopenia working groups, early recognition and intervention are key to proper management of sarcopenia (34, 39). If the same methodology of the current study is applied to the CT scans, obtained during diagnostic workup, patients with volumetric sarcopenia can be identified easily in the early phase of the treatment. For those, further muscle loss should be prevented by providing individualized consultation with a nutritional expert, adequate nutritional supplementation, and interventions with physical exercise, consisting of aerobic and resistance exercises during the course of primary treatment (40, 41).

To our knowledge, the current study is the first to report volumetric changes in both skeletal muscle and fat during primary treatment in patients with early cervical cancer. While significant decreases in waist skeletal muscle (median, −3.9%; *p* < 0.001) and total fat (median, −5.3%; *p* < 0.001) were observed in our volumetric measurement study, L3 SMI did not decrease significantly in the Taiwanese longitudinal study on locally advanced cervical cancer (17). Nevertheless, that study identified SMI loss >10% as an independent poor prognostic factor for OS. Among the treatment-related factors, we identified open RH, rather than minimally invasive RH, as an aggravating factor for the loss of skeletal muscle and total fat volumes. Compared with no adjuvant treatment, adjuvant (CC)RT was associated with the greater loss of total fat volume. In the study of Matsuoka et al. (18), anorexia and reduced food intake were frequently observed during postoperative care and at the time of adjuvant CCRT or RT (18). Similarly, we also observed high incidence of gastrointestinal toxicities during adjuvant (CC)RT. Especially, the presence of vomiting was significantly associated with the loss of total fat volume. Considering that gastrointestinal toxicities during adjuvant (CC)RT hinder patients’ food intake, such toxicities should be relieved by using antiemetics, antidiarrheal agents, and other drugs adequate to maintain body compositions (42, 43). Persistent or recurrent bowel obstruction, which might further aggravate malnutrition and loss of body weight, should be also managed properly (44).

Interestingly, 38.5% of the study population experienced gain of waist total fat volume, which was identified as a poor prognostic factor for PFS. While we conducted a volumetric approach, most previous studies have measured BMI and body weight change during cancer treatment. For example, Kroenke et al. reported relationship between weight gain after diagnosis and higher recurrence and mortality in breast cancer (45). Current evidence suggests that excessive visceral fat accumulation, also known as visceral obesity, is associated with adverse metabolic consequences, systemic inflammation, and cancer development and progression (46). In the current study, the patients who were initially volumetric sarcopenia and gained total fat during primary treatment were identified to have higher risk of disease recurrence than the others. Similar results were also observed in previous studies on ovarian cancer (31) and colorectal cancer (47). Worse PFS from the coexistence of sarcopenia and fat gain might be explained by the concept, sarcopenic obesity, known to affect the survival outcome of patients, which is equal to or greater than the sum of the respective risks of obesity and sarcopenia (48). As a possible explanation, researchers have indicated adipose stem cells from visceral and subcutaneous fat may promote the growth and migration of cancer cells (49). Therefore, initial sarcopenia patients should be cautious of excessive fat gain by avoiding excessive intake and lack of physical exercise (50, 51). It might be necessary to monitor body composition changes during the treatment courses.

Our study had several limitations. First, selection bias is one of the most problematic issues originating from the retrospective study design. For example, among the original study population, we further excluded patients who did not receive post-treatment CT scans to investigate the impact of changes in waist body composition on survival outcomes. We recognize that the excluded patients tended to belong to a favorable risk group, thus omitting adjuvant treatment after surgery. Second, the small sample size is also problematic. Owing to the small number of intraoperative and postoperative complications, the relationship between sarcopenia and complications related to surgery has not been reported. Further subgroup analyses by the administration of adjuvant treatment and detailed radiation methods were not performed because of the small number of recurrent and death cases. Third, we could not obtain BMI data after treatment or conduct further analysis based on the changes in BMI. Lastly, the precise underlying mechanisms for poor survival outcomes from volumetric sarcopenia and total fat gain could not be elucidated from the current study. Therefore, further cell line or animal level proof-of-concept studies are warranted.

## Conclusion

In conclusion, our study results demonstrate that waist volumetric SMI might be a prognostic biomarker for early cervical cancer. In particular, initial sarcopenia patients who gained body fat during primary treatment were at a high risk of disease recurrence. It is feasible to measure the waist volume of each body component and their longitudinal changes using the artificial intelligence-based volumetric tool. Further validation studies verifying our findings are warranted.

## Data Availability Statement

The raw data supporting the conclusions of this article will be made available by the authors, without undue reservation.

## Ethics Statement

The studies involving human participants were reviewed and approved by Institutional Review Board of Seoul National University Hospital (No. H-2012-061-117). Written informed consent for participation was not required for this study in accordance with the national legislation and the institutional requirements.

## Author Contributions

QH: investigation, data curation, formal analysis, and writing–original draft. SIK: methodology, investigation, data curation, formal analysis, and writing–original draft. SHY: resources, methodology, investigation, formal analysis, and writing–review and editing. TMK and JYC: investigation, validation, and writing–review and editing. H-CK and HJK: investigation, validation, and writing–review and editing. J-WK: conceptualization, resources, methodology, investigation, formal analysis, validation, supervision, and writing–review and editing. All authors contributed to the article and approved the submitted version.

## Conflict of Interest

SHY works in the MEDICALIP as a chief medical officer.

The remaining authors declare that the research was conducted in the absence of any commercial or financial relationships that could be construed as a potential conflict of interest.

## Publisher’s Note

All claims expressed in this article are solely those of the authors and do not necessarily represent those of their affiliated organizations, or those of the publisher, the editors and the reviewers. Any product that may be evaluated in this article, or claim that may be made by its manufacturer, is not guaranteed or endorsed by the publisher.
