# Supplementary figures and images for: Impact of Computed Tomography-Based, Artificial Intelligence-Driven Volumetric Sarcopenia on Survival Outcomes in Early Cervical Cancer
Source: Front Oncol. 2021 Sep 24;11:741071. doi: 10.3389/fonc.2021.741071 (PMC8499694; doi:10.3389/fonc.2021.741071)

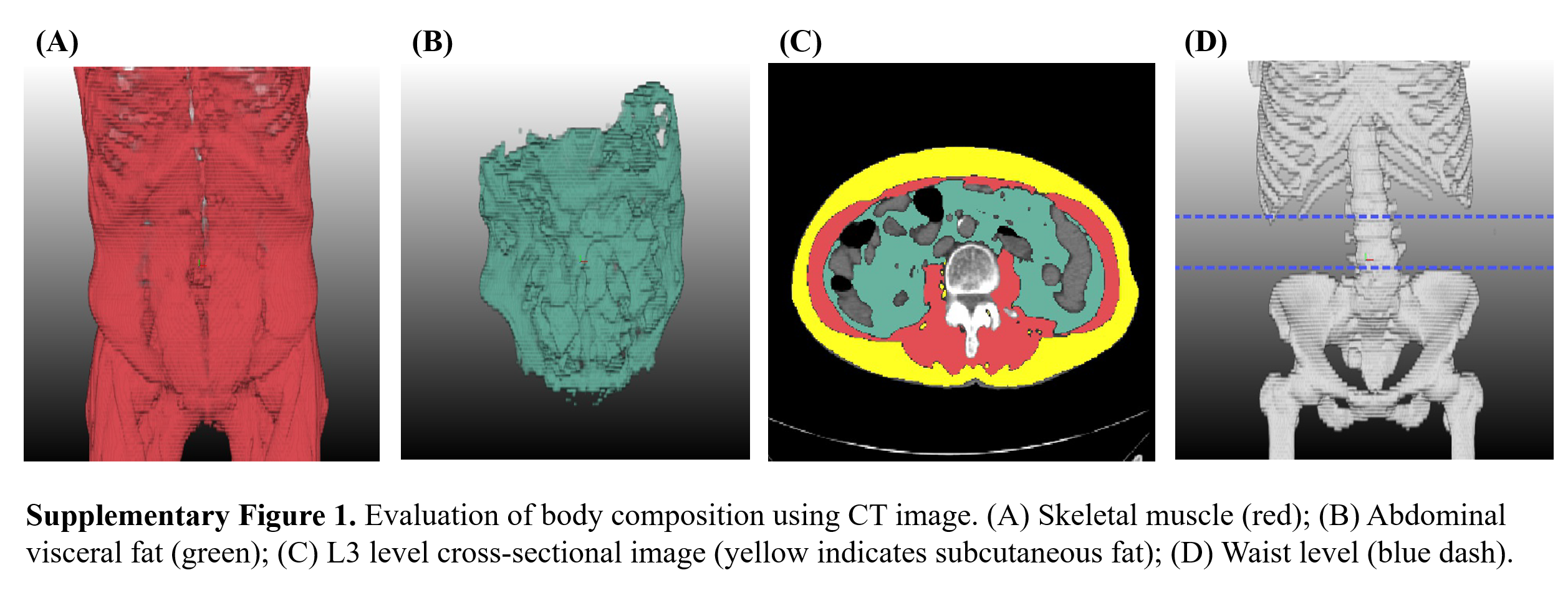

Supplement: Supplementary file 1 [file Image_1.tif]

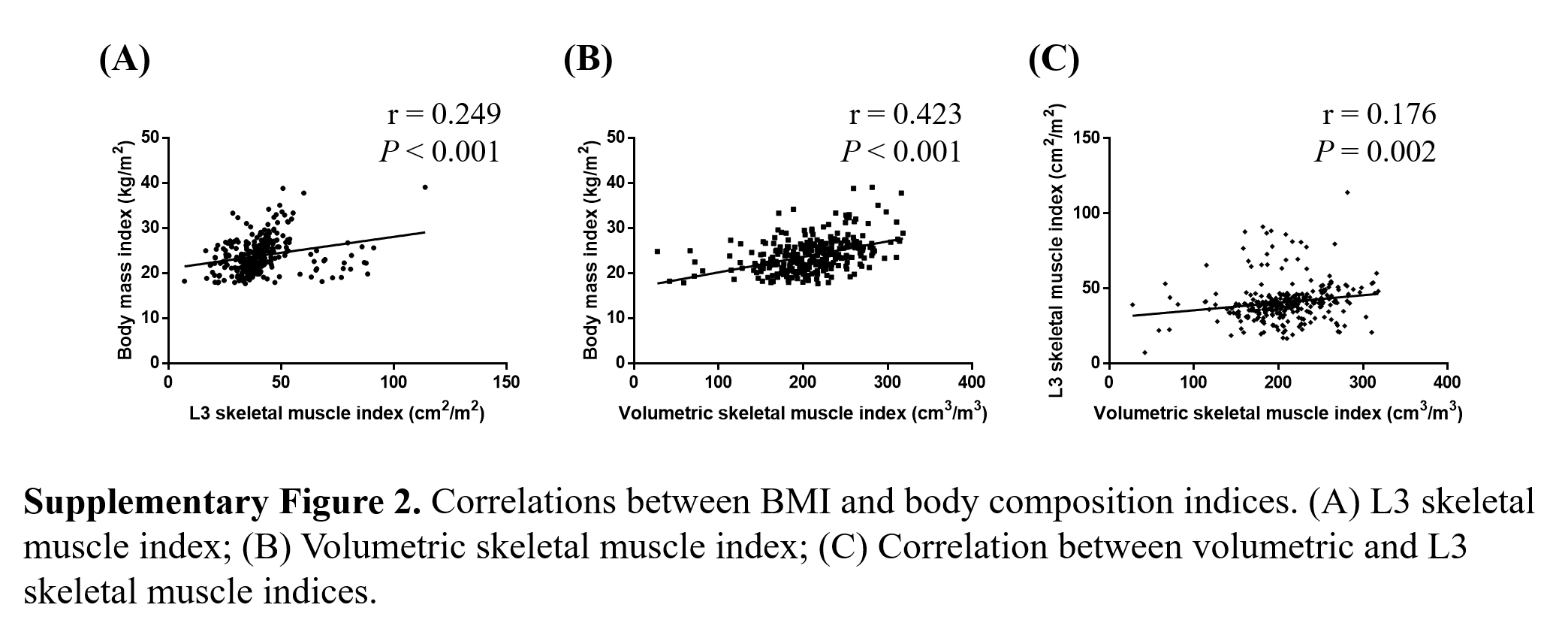

Supplement: Supplementary file 2 [file Image_2.tif]

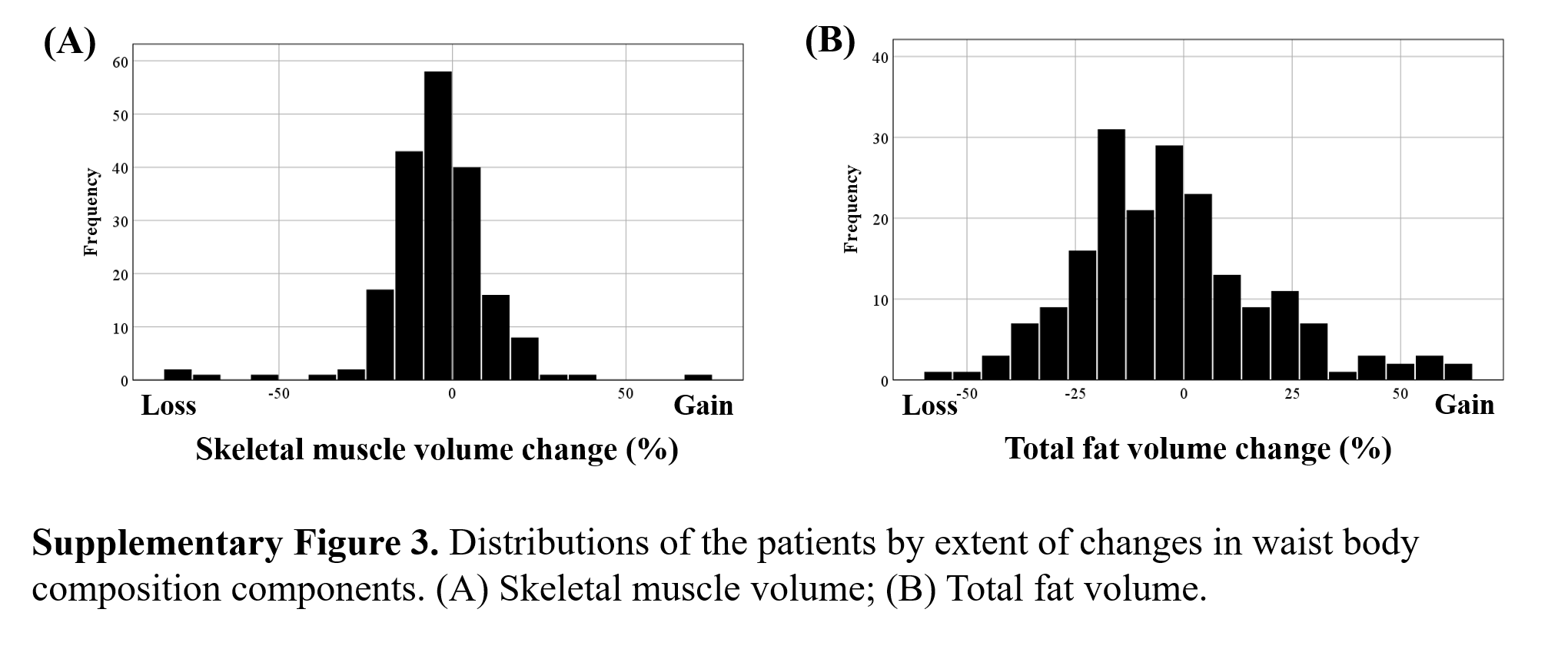

Supplement: Supplementary file 3 [file Image_3.tif]

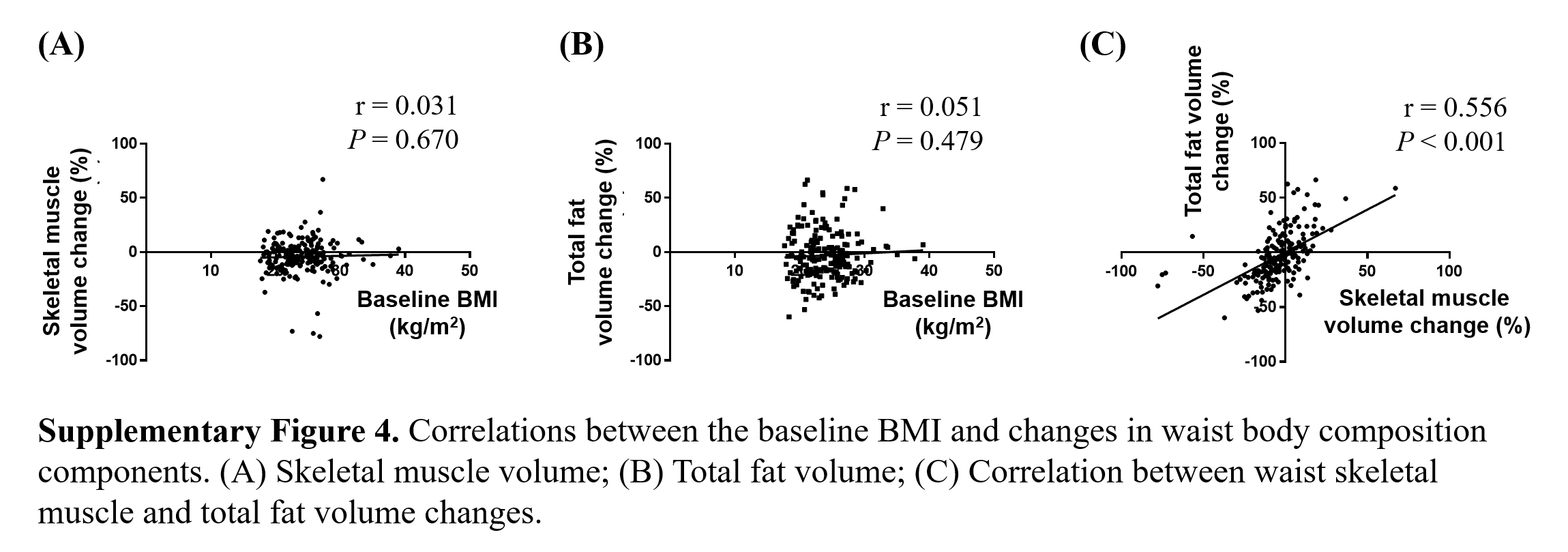

Supplement: Supplementary file 4 [file Image_4.tif]
