# Supplementary material for: Impact of Computed Tomography-Based, Artificial Intelligence-Driven Volumetric Sarcopenia on Survival Outcomes in Early Cervical Cancer
Source: Front Oncol. 2021 Sep 24;11:741071. doi: 10.3389/fonc.2021.741071 (PMC8499694; doi:10.3389/fonc.2021.741071)
Supplement: Supplementary file 5 [file Table_1.docx]

| **Supplementary Table 1.** Comparisons of radiation methods and gastrointestinal toxicity between volumetric sarcopenia and non-sarcopenia groups in patients who received adjuvant radiation therapy | | | | |  |  |
| --- | --- | --- | --- | --- | --- | --- |
| **Characteristics** | **All**  **(n=187, %)** | **Volumetric sarcopenia**  **(n=47, %)** | **Volumetric non-sarcopenia**  **(n=140, %)** | ***P*** | |  |
| Adjuvant treatment |  |  |  | 0.502 | |  |
| RT only | 30 (16.0) | 9 (19.1) | 21 (15.0) |  | |  |
| CCRT | 157 (84.0) | 38 (80.9) | 119 (85.0) |  | |  |
| EBRT planning |  |  |  | 0.006 | |  |
| 3D conformal RT | 116 (62.0) | 37 (78.7) | 79 (56.4) |  | |  |
| IMRT | 71 (38.0) | 10 (21.3) | 61 (43.6) |  | |  |
| Use of HDR-ICR |  |  |  | 0.310 | |  |
| No | 154 (82.4) | 41 (87.2) | 113 (80.7) |  | |  |
| Yes | 33 (17.6) | 6 (12.8) | 27 (19.3) |  | |  |
| Use of extended field RT^*^ |  |  |  | 0.454 | |  |
| No | 178 (95.2) | 46 (97.9) | 132 (94.3) |  | |  |
| Yes | 9 (4.8) | 1 (2.1) | 8 (5.7) |  | |  |
| Early termination of RT |  |  |  | 0.601 | |  |
| No | 182 (97.3) | 45 (95.7) | 137 (97.9) |  | |  |
| Yes^†^ | 5 (2.7) | 2 (4.3) | 3 (2.1) |  | |  |
| Gastrointestinal toxicity, any grade^‡^ |  |  |  |  | |  |
| Abdominal pain | 38 (20.3) | 12 (25.5) | 26 (18.6) | 0.305 | |  |
| Anorexia | 42 (22.5) | 8 (17.0) | 34 (24.3) | 0.302 | |  |
| Nausea | 103 (55.1) | 29 (61.7) | 74 (52.9) | 0.292 | |  |
| Vomiting | 36 (19.3) | 14 (29.8) | 22 (15.7) | 0.034 | |  |
| Diarrhea | 85 (45.5) | 28 (59.6) | 57 (40.7) | 0.025 | |  |
| Constipation | 59 (31.6) | 18 (38.3) | 41 (29.3) | 0.250 | |  |
| Abbreviations: CCRT, concurrent chemoradiation therapy; EBRT, external beam radiation therapy; HDR-ICR, high dose rate intracavitary radiotherapy; IMRT, intensity-modulated radiation therapy; RT, radiation therapy.  ^*^Boost on para-aortic area.  ^†^All five patients refused RT due to poor general condition during adjuvant treatment.  ^‡^Common Terminology Criteria for Adverse Events (CTCAE) version 5.0. | | | | | | |
