# Supplementary material for: Impact of Computed Tomography-Based, Artificial Intelligence-Driven Volumetric Sarcopenia on Survival Outcomes in Early Cervical Cancer
Source: Front Oncol. 2021 Sep 24;11:741071. doi: 10.3389/fonc.2021.741071 (PMC8499694; doi:10.3389/fonc.2021.741071)
Supplement: Supplementary file 6 [file Table_2.docx]

| **Supplementary Table 2.** Clinicopathologic characteristics of L3 sarcopenia and non-sarcopenia groups | | | |
| --- | --- | --- | --- |
| **Characteristics** | **L3 sarcopenia**  **(n=141, %)** | **L3 non-sarcopenia**  **(n=165, %)** | ***P*** |
| Age, years |  |  |  |
| Mean ± SD | 50.5 ± 11.1 | 52.2 ± 11.5 | 0.187 |
| BMI, kg/m^2^ |  |  |  |
| Median (IQR) | 22.1 (20.3−24.3) | 24.8 (22.2−27.0) | <0.001 |
| Underweight (<18.5) | 10 (7.1) | 2 (1.2) | 0.001 |
| Normal (18.5−22.9) | 77 (54.6) | 55 (33.3) |  |
| Overweight (23.0−24.9) | 26 (18.4) | 32 (19.4) |  |
| Obesity (≥25.0) | 28 (19.9) | 76 (46.1) |  |
| Surgical approach |  |  | 0.456 |
| Open | 63 (44.7) | 80 (48.5) |  |
| Laparoscopy | 60 (42.6)) | 71 (43.0) |  |
| Robot-assisted surgery | 18 (12.8) | 14 (8.5) |  |
| Conization | 41 (29.1) | 47 (28.5) | 0.909 |
| Histologic type |  |  | 0.796 |
| Squamous cell carcinoma | 107 (75.9) | 120 (72.7) |  |
| Adenocarcinoma | 28 (19.9) | 38 (23.0) |  |
| Adenosquamous carcinoma | 6 (4.3) | 7 (4.2) |  |
| 2009 FIGO stage |  |  | 0.943 |
| IB1 | 89 (63.1) | 107 (64.8) |  |
| IB2 | 23 (16.3) | 26 (15.8) |  |
| IIA1 | 9 (6.4) | 12 (7.3) |  |
| IIA2 | 20 (14.2) | 20 (12.1) |  |
| Radicality of hysterectomy |  |  | 0.858 |
| Type B | 12 (8.5) | 15 (9.1) |  |
| Type C | 129 (91.5) | 150 (90.9) |  |
| Para-aortic lymphadenectomy |  |  | 0.678 |
| No | 103 (73.0) | 117 (70.9) |  |
| Sampling/Dissection | 38 (27.0) | 48 (29.1) |  |
| Clinical cervical tumor size^*^, mm |  |  |  |
| Median (IQR) | 30.0 (10.0−41.0) | 25.0 (10.0−40.0) | 0.565 |
| <20 | 51 (36.2) | 58 (35.2) | 0.634 |
| ≥20 and <40 | 47 (33.3) | 63 (38.2) |  |
| ≥40 | 43 (30.5) | 44 (26.7) |  |
| Pathologic risk factors |  |  |  |
| Parametrial invasion | 31 (22.0) | 31 (18.8) | 0.488 |
| Lymph node metastasis | 34 (24.1) | 51 (30.9) | 0.186 |
| Resection margin involvement | 16 (11.3) | 14 (8.5) | 0.401 |
| LVSI | 73 (51.8) | 81 (49.1) | 0.640 |
| Deep one-third stromal invasion | 74 (52.5) | 87 (52.7) | 0.966 |
| Risk group |  |  | 0.758 |
| Low-risk | 58 (41.1) | 61 (37.0) |  |
| Intermediate-risk | 31 (22.0) | 39 (23.6) |  |
| High-risk | 52 (36.9) | 65 (39.4) |  |
| Adjuvant treatment |  |  | 0.678 |
| No | 55 (39.0) | 64 (38.8) |  |
| RT only | 16 (11.3) | 14 (8.5) |  |
| CCRT | 70 (49.6) | 87 (52.7) |  |
| Abbreviations: BMI, body mass index; CCRT, concurrent chemoradiation therapy; FIGO, International Federation of Gynecology and Obstetrics; IQR, interquartile range; LVSI, lymphovascular space invasion; RT, radiation therapy; SD, standard deviation.  ^*^Measured by either colposcopic examination or pre-treatment magnetic resonance imaging. | | | |
|  | |  |  |
