# Supplementary material for: Impact of Computed Tomography-Based, Artificial Intelligence-Driven Volumetric Sarcopenia on Survival Outcomes in Early Cervical Cancer
Source: Front Oncol. 2021 Sep 24;11:741071. doi: 10.3389/fonc.2021.741071 (PMC8499694; doi:10.3389/fonc.2021.741071)
Supplement: Supplementary file 7 [file Table_3.docx]

| **Supplementary Table 3.** Comparisons of clinicopathologic characteristics between patients with and without post-treatment CT scans | | | |
| --- | --- | --- | --- |
| **Characteristics** | **Without post-treatment**  **CT scans (n=114, %)** | **With post-treatment**  **CT scans (n=192, %)** | ***P*** |
| Age, years |  |  |  |
| Mean ± SD | 51.6 ± 11.4 | 51.4 ± 11.3 | 0.905 |
| BMI, kg/m^2^ |  |  |  |
| Median (IQR) | 22.8 (21.2−25.5) | 23.6 (21.2−26.2) | 0.404 |
| Underweight (<18.5) | 5 (4.4) | 7 (3.6) | 0.413 |
| Normal (18.5−22.9) | 53 (46.5) | 79 (41.1) |  |
| Overweight (23.0−24.9) | 24 (21.1) | 34 (17.7) |  |
| Obesity (≥25.0) | 32 (28.1) | 72 (37.5) |  |
| Surgical approach |  |  | 0.211 |
| Open | 48 (42.1) | 95 (49.5) |  |
| Minimally invasive surgery | 66 (57.9) | 97 (50.5) |  |
| Conization | 35 (30.7) | 53 (27.6) | 0.563 |
| Histologic type |  |  | 0.217 |
| Squamous cell carcinoma | 80 (70.2) | 147 (76.6) |  |
| Non-squamous cell carcinoma | 34 (29.8) | 45 (23.4) |  |
| 2009 FIGO stage |  |  | 0.379 |
| IB1 | 80 (70.2) | 116 (60.4) |  |
| IB2 | 16 (14.0) | 33 (17.2) |  |
| IIA1 | 6 (5.3) | 15 (7.8) |  |
| IIA2 | 12 (10.5) | 28 (14.6) |  |
| Radicality of hysterectomy |  |  | 0.659 |
| Type B | 9 (7.9) | 18 (9.4) |  |
| Type C | 105 (92.1) | 174 (90.6) |  |
| Para-aortic lymphadenectomy |  |  | 0.017 |
| No | 91 (79.8) | 129 (67.2) |  |
| Sampling/Dissection | 23 (20.2) | 63 (32.8) |  |
| Clinical cervical tumor size^*^, mm |  |  |  |
| Median (IQR) | 20.0 (10.0−37.3) | 30.0 (13.5−41.0) | 0.026 |
| <20 | 48 (42.1) | 61 (31.8) | 0.089 |
| ≥20 and <40 | 41 (36.0) | 69 (35.9) |  |
| ≥40 | 25 (21.9) | 62 (32.3) |  |
| Pathologic risk factors |  |  |  |
| Parametrial invasion | 17 (14.9) | 45 (23.4) | 0.073 |
| Lymph node metastasis | 23 (20.2) | 62 (32.3) | 0.022 |
| Resection margin involvement | 9 (7.9) | 21 (10.9) | 0.387 |
| LVSI | 55 (48.2) | 99 (51.6) | 0.575 |
| Deep one-third stromal invasion | 55 (48.2) | 106 (55.2) | 0.238 |
| Risk group |  |  | 0.019 |
| Low-risk | 52 (45.3) | 67 (34.9) |  |
| Intermediate-risk | 30 (26.3) | 40 (20.8) |  |
| High-risk | 32 (28.1) | 85 (44.3) |  |
| Adjuvant treatment |  |  | <0.001 |
| No | 53 (46.5) | 66 (34.4) |  |
| RT only | 22 (19.3) | 8 (4.2) |  |
| CCRT | 39 (34.2) | 118 (61.5) |  |
| Abbreviations: BMI, body mass index; CCRT, concurrent chemoradiation therapy; FIGO, International Federation of Gynecology and Obstetrics; IQR, interquartile range; LVSI, lymphovascular space invasion; RT, radiation therapy; SD, standard deviation.  ^*^Measured by either colposcopic examination or pre-treatment magnetic resonance imaging. | | | |
