# Supplementary material for: Impact of Computed Tomography-Based, Artificial Intelligence-Driven Volumetric Sarcopenia on Survival Outcomes in Early Cervical Cancer
Source: Front Oncol. 2021 Sep 24;11:741071. doi: 10.3389/fonc.2021.741071 (PMC8499694; doi:10.3389/fonc.2021.741071)
Supplement: Supplementary file 8 [file Table_4.docx]

| **Supplementary Table 4.** Changes in waist body composition | | | | | | |
| --- | --- | --- | --- | --- | --- | --- |
| **Characteristics** | | **N** | **Pre-treatment** | **Post-treatment** | **Change (%)** | ***P*** |
| All patients | | 192 |  |  |  |  |
| Skeletal muscle index, cm^3^/m^3^ | |  | 210.1 (186.6−238.0) | 203.7 (177.6−230.2) | -3.9 (-11.0−3.7) | <0.001 |
| Total fat index, cm^3^/m^3^ | |  | 508.0 (351.4−678.7) | 385.9 (277.1−510.6) | -5.3 (-17.6−8.0) | <0.001 |
| **Characteristics** | **N** | | **Skeletal muscle volume change (%)** | ***P*** | **Total fat volume change (%)** | ***P*** |
| Baseline BMI, kg/m^2^ |  | |  | 0.454 |  | 0.742 |
| Underweight-normal (<23.0) | 86 | | -4.2 (-12.1−2.4) |  | -5.3 (-19.4−10.2) |  |
| Overweight (23.0−24.9) | 34 | | -3.8 (-9.0−5.2) |  | -3.3 (-16.5−14.7) |  |
| Obesity (≥25.0) | 72 | | -4.8 (-10.1−6.0) |  | -5.8 (-17.9−5.2) |  |
| Baseline volumetric sarcopenia |  | |  | 0.003 |  | 0.011 |
| Yes | 39 | | 1.2 (-8.8−16.5) |  | 6.0 (-16.1−17.2) |  |
| No | 153 | | -4.5 (-11.3−2.2) |  | -6.7 (-18.3−4.1) |  |
| Surgical approach |  | |  | 0.005 |  | <0.001 |
| Open | 95 | | -7.5 (-13.3−1.2) |  | -12.9 (-21.3−1.5) |  |
| Laparoscopy | 71 | | -2.2 (-7.6−5.1) |  | -2.4 (-11.6−10.7) |  |
| Robot-assisted surgery | 26 | | -1.0 (-10.4−9.8) |  | 4.7 (-6.4−24.2) |  |
| 2009 FIGO stage |  | |  | 0.296 |  | 0.002 |
| IB1 | 116 | | -3.6 (-10.1−3.6) |  | -2.9 (-15.8−12.7) |  |
| IB2 | 33 | | -4.0 (-10.2−6.3) |  | -5.8 (-18.3−7.0) |  |
| IIA1 | 15 | | 0.0 (-9.2−8.2) |  | -2.4 (-12.9−9.3) |  |
| IIA2 | 28 | | -6.3 (-16.7−1.5) |  | -17.1 (-27.3−-7.2) |  |
| Risk group |  | |  | 0.155 |  | 0.104 |
| Low-risk | 67 | | -2.5 (-8.8−3.1) |  | -2.5 (-10.6−9.0) |  |
| Intermediate-risk | 40 | | -6.6 (-13.7−0.9) |  | -11.1 (-19.0−5.6) |  |
| High-risk | 85 | | -4.2 (-11.6−5.3) |  | -7.4 (-19.7−9.0) |  |
| Adjuvant treatment |  | |  | 0.168 |  | 0.099 |
| No^*^ | 66 | | -2.4 (-8.9−3.5) |  | -2.3 (-13.4−9.1) |  |
| RT only^†^ | 8 | | 0.0 (-10.4−17.1) | ^*,†^0.577 | -5.0 (-21.5−17.3) | ^*,†^0.577 |
| CCRT^‡^ | 118 | | -4.8 (-12.4−3.2) | ^*,‡^0.102 | -8.7 (-19.3−6.9) | ^*,‡^0.032 |
| Presented with median value with interquartile range.  Abbreviations: BMI, body mass index; CCRT, concurrent chemoradiation therapy; FIGO, International Federation of Gynecology and Obstetrics; RT, radiation therapy. | | | | | | |
