# Supplementary material for: Impact of Computed Tomography-Based, Artificial Intelligence-Driven Volumetric Sarcopenia on Survival Outcomes in Early Cervical Cancer
Source: Front Oncol. 2021 Sep 24;11:741071. doi: 10.3389/fonc.2021.741071 (PMC8499694; doi:10.3389/fonc.2021.741071)
Supplement: Supplementary file 9 [file Table_5.docx]

| **Supplementary Table 5.** Changes in waist body composition by radiation methods and gastrointestinal toxicity among patients who received adjuvant radiation therapy | | | | | |  |
| --- | --- | --- | --- | --- | --- | --- |
| **Characteristics** | **N** | **Skeletal muscle volume change (%)** | ***P*** | **Total fat volume change (%)** | ***P*** |  |
| All patients | 126 | -4.5 (-12.1−3.8) | <0.001 | -8.5 (-19.3−6.9) | <0.001 |  |
| Adjuvant treatment |  |  | 0.246 |  | 0.689 |  |
| RT only | 8 | -0.0 (-10.4−17.1) |  | -5.0 (-21.5−17.3) |  |  |
| CCRT | 118 | -4.8 (-12.4−3.2) |  | -8.7 (-19.3−6.9) |  |  |
| EBRT planning |  |  | 0.538 |  | 0.795 |  |
| 3D conformal RT | 73 | -4.0 (-12.0−4.9) |  | -8.3 (-18.8−5.9) |  |  |
| IMRT | 53 | -7.0 (-12.6−2.6) |  | -9.1 (-19.8−10.5) |  |  |
| Use of HDR-ICR |  |  | 0.707 |  | 0.739 |  |
| No | 101 | -4.5 (-12.0−3.5) |  | -8.7 (-19.2−8.7) |  |  |
| Yes | 25 | -5.2 (-12.6−4.9) |  | -6.8 (-22.4−2.4) |  |  |
| Use of extended field RT^*^ |  |  | 0.645 |  | 0.131 |  |
| No | 118 | -4.5 (-12.0−3.5) |  | -7.1 (-19.1−7.9) |  |  |
| Yes | 8 | -9.5 (-15.6−7.1) |  | -18.0 (-45.2−-6.3) |  |  |
| Abdominal pain, any grade^†^ |  |  |  |  |  |  |
| No | 101 | -5.2 (-11.8−3.6) |  | -5.6 (-18.5−7.3) |  |  |
| Yes | 25 | -4.0 (-16.8−5.8) |  | -15.4 (-26.0−6.0) |  |  |
| Anorexia, any grade^†^ |  |  | 0.937 |  | 0.969 |  |
| No | 94 | -4.8 (-13.1−4.7) |  | -7.1 (-19.5−6.9) |  |  |
| Yes | 32 | -4.2 (-11.9−1.2) |  | -9.8 (-18.3−9.7) |  |  |
| Nausea, any grade^†^ |  |  | 0.541 |  | 0.906 |  |
| No | 58 | -4.4 (-11.6−5.2) |  | -8.9 (-18.9−6.0) |  |  |
| Yes | 68 | -5.3 (-13.1−2.7) |  | -5.7 (-20.0−9.8) |  |  |
| Vomiting, any grade^†^ |  |  | 0.162 |  | 0.049 |  |
| No | 103 | -4.2 (-12.0−4.8) |  | -5.6 (-18.5−7.9) |  |  |
| Yes | 23 | -7.6 (-13.2−-1.7) |  | -17.1 (-26.3−-2.4) |  |  |
| Diarrhea, any grade^†^ |  |  | 0.668 |  | 0.801 |  |
| No | 72 | -5.9 (-13.1−4.4) |  | -5.6 (-19.2−6.4) |  |  |
| Yes | 54 | -4.1 (-11.6−3.5) |  | -9.8 (-19.8−7.9) |  |  |
| Constipation, any grade^†^ |  |  | 0.645 |  | 0.437 |  |
| No | 82 | -4.5 (-11.6−3.5) |  | -5.7 (-20.6−13.3) |  |  |
| Yes | 44 | -5.0 (-15.6−4.6) |  | -11.4 (-18.4−4.0) |  |  |
| Presented with median value with interquartile range.  Abbreviations: CCRT, concurrent chemoradiation therapy; EBRT, external beam radiation therapy; HDR-ICR, high dose rate intracavitary radiotherapy; IMRT, intensity-modulated radiation therapy; RT, radiation therapy.  ^*^Boost on para-aortic area.  ^†^Common Terminology Criteria for Adverse Events (CTCAE) version 5.0. | | | | | | |
