# Supplementary material for: Impact of Computed Tomography-Based, Artificial Intelligence-Driven Volumetric Sarcopenia on Survival Outcomes in Early Cervical Cancer
Source: Front Oncol. 2021 Sep 24;11:741071. doi: 10.3389/fonc.2021.741071 (PMC8499694; doi:10.3389/fonc.2021.741071)
Supplement: Supplementary file 10 [file Table_6.docx]

| **Supplementary Table 6.** Clinicopathologic characteristics of total fat gain and loss groups | | | |
| --- | --- | --- | --- |
| **Characteristics** | **Total fat gain**  **(n=74, %)** | **Total fat loss**  **(n=118, %)** | ***P*** |
| Age, years |  |  |  |
| Mean ± SD | 51.2 ± 11.3 | 51.4 ± 11.4 | 0.909 |
| BMI, kg/m^2^ |  |  |  |
| Median (IQR) | 23.6 (21.2−26.4) | 23.6 (21.2−26.0) | 0.949 |
| Underweight (<18.5) | 3 (4.1) | 4 (3.4) | 0.950 |
| Normal (18.5−22.9) | 31 (41.9) | 48 (40.7) |  |
| Overweight (23.0−24.9) | 14 (18.9) | 20 (16.9) |  |
| Obesity (≥25.0) | 26 (35.1) | 46 (39.0) |  |
| Surgical approach |  |  | 0.001 |
| Open | 25 (33.8) | 70 (59.3) |  |
| Laparoscopy | 32 (43.2) | 39 (33.1) |  |
| Robot-assisted surgery | 17 (23.0) | 9 (7.6) |  |
| Conization | 23 (31.1) | 30 (25.4) | 0.393 |
| Histologic type |  |  | 0.206 |
| Squamous cell carcinoma | 60 (81.1) | 87 (73.7) |  |
| Adenocarcinoma | 14 (18.9) | 27 (22.9) |  |
| Adenosquamous carcinoma | 0 | 4 (3.4) |  |
| 2009 FIGO stage |  |  | 0.087 |
| IB1 | 50 (67.6) | 66 (55.9) |  |
| IB2 | 12 (16.2) | 21 (17.8) |  |
| IIA1 | 7 (9.5) | 8 (6.8) |  |
| IIA2 | 5 (6.8) | 23 (19.5) |  |
| Radicality of hysterectomy |  |  | 0.589 |
| Type B | 8 (10.8) | 10 (8.5) |  |
| Type C | 66 (89.2) | 108 (91.5) |  |
| Para-aortic lymphadenectomy |  |  | 0.001 |
| No | 60 (81.1) | 69 (58.5) |  |
| Sampling/Dissection | 14 (18.9) | 49 (41.5) |  |
| Clinical cervical tumor size^*^, mm |  |  |  |
| Median (IQR) | 25.0 (10.0−40.1) | 31.5 (16.5−42.0) | 0.112 |
| <20 | 29 (39.2) | 32 (27.1) | 0.195 |
| ≥20 and <40 | 25 (33.8) | 44 (37.3) |  |
| ≥40 | 20 (27.0) | 42 (35.6) |  |
| Pathologic risk factors |  |  |  |
| Parametrial invasion | 14 (18.9) | 31 (26.3) | 0.242 |
| Lymph node metastasis | 23 (31.1) | 39 (33.1) | 0.776 |
| Resection margin involvement | 4 (5.4) | 17 (14.4) | 0.052 |
| LVSI | 34 (45.9) | 65 (55.1) | 0.217 |
| Deep one-third stromal invasion | 36 (48.6) | 70 (59.3) | 0.148 |
| Risk group |  |  | 0.403 |
| Low-risk | 30 (40.5) | 37 (31.4) |  |
| Intermediate-risk | 15 (20.3) | 25 (21.2) |  |
| High-risk | 29 (39.2) | 56 (47.5) |  |
| Adjuvant treatment |  |  | 0.217 |
| No | 31 (41.9) | 35 (29.7) |  |
| RT only | 3 (4.1) | 5 (4.2) |  |
| CCRT | 40 (54.1) | 78 (66.1) |  |
| Abbreviations: BMI, body mass index; CCRT, concurrent chemoradiation therapy; FIGO, International Federation of Gynecology and Obstetrics; IQR, interquartile range; LVSI, lymphovascular space invasion; RT, radiation therapy; SD, standard deviation.  ^*^Measured by either colposcopic examination or pre-treatment magnetic resonance imaging. | | | |
|  | |  |  |
