# Supplementary material for: Impact of Computed Tomography-Based, Artificial Intelligence-Driven Volumetric Sarcopenia on Survival Outcomes in Early Cervical Cancer
Source: Front Oncol. 2021 Sep 24;11:741071. doi: 10.3389/fonc.2021.741071 (PMC8499694; doi:10.3389/fonc.2021.741071)
Supplement: Supplementary file 11 [file Table_7.docx]

| **Supplementary Table 7.** Clinicopathologic characteristics by baseline volumetric sarcopenia and waist fat change | | | | | |
| --- | --- | --- | --- | --- | --- |
| **Characteristics** | **Others**  **(n=170, %)** | | **Sarcopenia plus**  **fat gain (n=22, %)** | | ***P*** |
| Age, years |  | |  | |  |
| Mean ± SD | 51.2 ± 11.3 | | 51.4 ± 11.4 | | 0.330 |
| BMI, kg/m^2^ |  | |  | |  |
| Median (IQR) | 23.6 (21.4−26.0) | | 22.5 (20.6−26.7) | | 0.750 |
| Underweight (<18.5) | 6 (3.5) | | 1 (4.5) | | 0.386 |
| Normal (18.5−22.9) | 68 (40.0) | | 11 (50.0) | |  |
| Overweight (23.0−24.9) | 33 (19.4) | | 1 (4.5) | |  |
| Obesity (≥25.0) | 63 (37.1) | | 9 (40.9) | |  |
| Surgical approach |  | |  | | 0.787 |
| Open | 85 (50.0) | | 10 (45.5) | |  |
| Laparoscopy | 63 (37.1) | | 8 (36.4) | |  |
| Robot-assisted surgery | 22 (12.9) | | 4 (18.2) | |  |
| Conization | 50 (29.4) | | 3 (13.6) | | 0.119 |
| Histologic type |  | |  | | 0.696 |
| Squamous cell carcinoma | 129 (75.9) | | 18 (81.8) | |  |
| Adenocarcinoma | 37 (21.8) | | 4 (18.2) | |  |
| Adenosquamous carcinoma | 4 (2.4) | | 0 | |  |
| 2009 FIGO stage |  | |  | | 0.738 |
| IB1 | 102 (60.0) | | 14 (36.6) | |  |
| IB2 | 31 (18.2) | | 2 (9.1) | |  |
| IIA1 | 13 (7.6) | | 2 (9.1) | |  |
| IIA2 | 24 (14.1) | | 4 (18.2) | |  |
| Radicality of hysterectomy |  | |  | | 0.039 |
| Type B | 13 (7.6) | | 5 (22.7) | |  |
| Type C | 157 (92.4) | | 17 (77.3) | |  |
| Para-aortic lymphadenectomy |  | |  | | 0.120 |
| No | 111 (65.3) | | 18 (81.8) | |  |
| Sampling/Dissection | 59 (34.7) | | 4 (18.2) | |  |
| Clinical cervical tumor size^*^, mm |  | |  | |  |
| Median (IQR) | 30.0 (12.0−41.3) | | 29.0 (18.3−41.3) | | 0.943 |
| <20 | 55 (32.4) | | 6 (27.3) | | 0.613 |
| ≥20 and <40 | 59 (34.7) | | 10 (45.5) | |  |
| ≥40 | 56 (32.9) | | 6 (27.3) | |  |
| Pathologic risk factors |  | |  | |  |
| Parametrial invasion | 38 (22.4) | | 7 (31.8) | | 0.324 |
| Lymph node metastasis | 54 (31.8) | | 8 (36.4) | | 0.664 |
| Resection margin involvement | 19 (11.2) | | 2 (9.1) | | >0.999 |
| LVSI | 89 (52.4) | | 10 (45.5) | | 0.542 |
| Deep one-third stromal invasion | 94 (55.3) | | 12 (54.5) | | 0.947 |
| Risk group |  | |  | | 0.665 |
| Low-risk | 59 (34.7) | | 8 (36.4) | |  |
| Intermediate-risk | 37 (21.8) | | 3 (13.6) | |  |
| High-risk | 74 (43.5) | | 11 (50.0) | |  |
| Adjuvant treatment |  | |  | | 0.971 |
| No | 58 (34.1) | | 8 (36.4) | |  |
| RT only | 7 (4.1) | | 1 (4.5) | |  |
| CCRT | 105 (61.8) | | 13 (59.1) | |  |
| Abbreviations: BMI, body mass index; CCRT, concurrent chemoradiation therapy; FIGO, International Federation of Gynecology and Obstetrics; IQR, interquartile range; LVSI, lymphovascular space invasion; RT, radiation therapy; SD, standard deviation.  ^*^Measured by either colposcopic examination or pre-treatment magnetic resonance imaging. | | | | | |
|  | |  | |  | |
